# Use of mixed-treatment-comparison methods in estimating efficacy of treatments for heavy menstrual bleeding

**DOI:** 10.1186/2047-783X-18-17

**Published:** 2013-06-21

**Authors:** David C Hoaglin, Anna Filonenko, Mark E Glickman, Radek Wasiak, Risha Gidwani

**Affiliations:** 1 United BioSource Corporation, 26-28 Hammersmith Grove, London, W6 7HA, UK; 2 Consulting Statistician, 73 Hickory Road, Sudbury, MA, 01776, USA; 3 Bayer Pharma AG, BSP-GMACS-GHEOR-WH/DI, Berlin, Germany; 4 Center for Health Quality, Outcomes & Economics Research, Boston University, Edith Nourse Rogers Memorial Hospital (152), Bldg 70, 200 Springs Road, Bedford, MA, 01730, USA; 5 VA Health Economics Resource Center, 795 Willow Road 152 MPD, Menlo Park, CA, 94025, USA

## Abstract

**Background:**

A variety of pharmacological and surgical treatments have been developed for heavy menstrual bleeding (HMB), which can have negative physical, social, psychological, and economic consequences. We conducted a systematic literature review and mixed-treatment-comparison (MTC) meta-analysis of available data from randomized controlled trials (RCTs) to derive estimates of efficacy for 8 classes of treatments for HMB, to inform health-economic analysis and future studies.

**Methods:**

A systematic review identified RCTs that reported data on menstrual blood loss (MBL) at baseline and one or more follow-up times. Eight treatment classes were considered: COCs, danazol, endometrial ablation, LNG-IUS, placebo, progestogens given for less than 2 weeks out of 4 during the menstrual cycle, progestogens given for close to 3 weeks out of 4, and TXA. The primary measure of efficacy was the proportion of women who achieved MBL < 80 mL per cycle (month), as measured by the alkaline hematin method. A score less than 100 on an established pictorial blood-loss assessment chart (PBAC) was considered an acceptable substitute for MBL < 80 mL. Estimates of efficacy by treatment class and time were obtained from a Bayesian MTC model. The model also included effects for treatment class, study, and the combination of treatment class and study and an adjustment for baseline mean MBL. Several methodological challenges complicated the analysis. Some trials reported various summary statistics for MBL or PBAC, requiring estimation (with less precision) of % MBL < 80 mL or % PBAC < 100. Also, reported follow-up times varied substantially.

**Results:**

The evidence network involved 34 RCTs, with follow-up times from 1 to 36 months. Efficacy at 3 months of follow-up (estimated as the posterior median) ranged from 87.5% for the levonorgestrel-releasing intrauterine system (LNG-IUS) to 14.2% for progestogens administered for less than 2 weeks out of 4 in the menstrual cycle. The 95% credible intervals for most estimates were quite wide, mainly because of the limited evidence for many combinations of treatment class and follow-up time and the uncertainty from estimating % MBL < 80 mL or % PBAC < 100 from summary statistics.

**Conclusions:**

LNG-IUS and endometrial ablation are very efficacious in treating HMB. The study yielded useful insights on using MTC in sparse evidence networks. Diversity of outcome measures and follow-up times in the HMB literature presented considerable challenges. The Bayesian credible intervals reflected the various sources of uncertainty.

## Background

Heavy menstrual bleeding (HMB) is a common but empirically challenging health condition. Although HMB is defined as menstrual flow exceeding 80 milliliters (mL) of blood loss per menstrual cycle that cannot be explained by organic pathology or medical illness, [1] the diagnosis is subjective, at least initially, and women vary in their perceptions of what is acceptable blood loss and when to seek help.

In surveys, between 13% and 52% of women report having HMB, depending on the country, age group, and definition of HMB [2-5]. However, fewer than 1 in 5 women who met criteria for HMB in England had sought treatment from their general practitioner [6]. Another study found that only one third of women referred to a gynecology clinic in Scotland for HMB actually had a mean menstrual blood loss (MBL) greater than 80 mL when this was formally measured, [7] suggesting a more conservative prevalence of HMB between 11% and 13% [2].

HMB can have negative physical, social, psychological, and economic consequences. MBL greater than 80 mL is likely to lead to anemia, [1] which affected around one quarter of women hospitalized for HMB in one US study [8]. HMB also impairs a woman’s quality of life [2] and is associated with a reduced likelihood of being employed [5].

A variety of pharmacological and surgical treatments aim to reduce MBL or eliminate menstruation altogether. Classes of treatments include combined oral contraceptives (COCs), tranexamic acid (TXA), oral or injectable progestogens, danazol, the levonorgestrel-releasing intrauterine system (LNG-IUS), and endometrial ablation or resection. An informed choice requires information on the clinical efficacy of relevant treatment options. In previous systematic reviews the evidence base on this topic has been weak, with few direct comparisons among treatment options, [9-12] leading to uncertainty about the overall comparative effectiveness of the most commonly used treatments. We therefore conducted a systematic literature review and mixed-treatment-comparison (MTC) meta-analysis to inform the development of a microsimulation model that assessed the cost-effectiveness of pharmacological interventions and endometrial ablation for HMB [13]. A type of network meta-analysis, MTCs combine information from direct and indirect comparisons of interventions, to allow estimation of the relative efficacy of interventions that have not been directly compared in head-to-head studies [14]. Our focus, however, was on estimating absolute efficacy (for use in the microsimulation model), rather than on assessing relative efficacy. During the review and analysis we identified a number of methodological challenges that should inform future research.

## Methods

### Treatments compared

We considered eight treatment classes: COCs, danazol, endometrial ablation, LNG-IUS, placebo, progestogens given for less than 2 weeks out of 4 during the menstrual cycle, progestogens given for close to 3 weeks out of 4, and TXA. We made no distinction between first- and second-generation endometrial ablation techniques.

### Literature search

**Figure 1:**
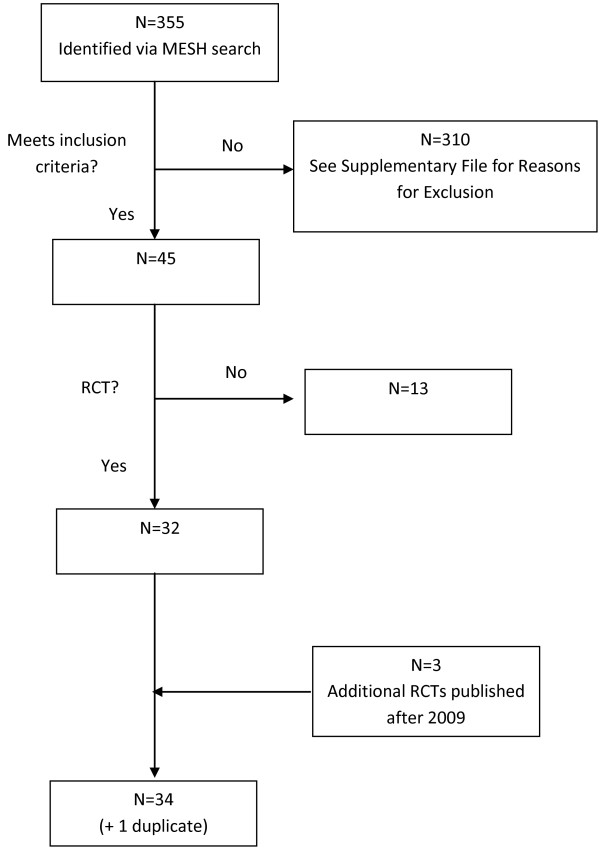
Summary of the literature search process.

Figure 1 summarizes the literature search process. Most of the articles that provided data for the present analysis were identified as part of a systematic review of the literature on HMB, covering the period 1966–2009. That review included a replication and update of a literature review previously employed for the National Institute for Health and Clinical Excellence (NICE) HMB guideline [2]. The search used the Cochrane Library, MEDLINE, EMBASE, CINAHL, PsychINFO, and the National Health Service (NHS) Economic Evaluation Database and included manual searches of the bibliographies of all review articles, as well as ad hoc internet searches for key treatment-related terms.

The titles and abstracts of the resulting items were reviewed against predefined inclusion and exclusion criteria. (See Additional file 1 for the inclusion and exclusion criteria for the full search and for studies of efficacy.) Items that had not been excluded were reviewed in full text, and data were extracted from items that passed this second level of review. Additional publications in the period 2009–2011 were identified through a manual search. Extracted data were reviewed for accuracy and completeness by an independent researcher.

Articles were assessed for quality using the Centre for Evidence Based Medicine (CEBM), (University of Oxford) quality score and the Grading of Recommendations Assessment, Development and Evaluation (GRADE) score [15].

### Efficacy measures

The primary measure of efficacy was the proportion of women who achieved MBL < 80 mL per cycle (month), as measured by the alkaline hematin method [16,17]. This measure allows objective estimation of blood loss, provided the patient accurately collects all sanitary material and submits it for analysis. As a less burdensome substitute, several researchers have developed pictorial charts, on which the patient records the blood loss by its appearance on various types of sanitary material; an investigator then uses a scoring system to calculate a numerical score for the cycle. The most widely used is the pictorial blood-loss assessment chart (PBAC) developed by Higham et al. [18], for which a score less than 100 is considered equivalent to MBL < 80 mL.

The choice of MBL as the efficacy measure was determined mainly by the outcomes reported in the randomized controlled trials (RCTs). A patient-reported outcome would enhance generalizability and relevance of the results, but inconsistent use of different scales with unclear psychometric properties for the HMB population made such a measure infeasible [19].

We extracted data if authors used the alkaline hematin method to measure blood loss, or if subjects used the Higham PBAC chart to assess their blood loss. Studies in which MBL was objectively measured could report the mean or median MBL with an accompanying measure of spread, or the proportion of women who achieved MBL < 80 mL per menstrual cycle at a particular follow-up time. The latter data were directly used as inputs for the analysis. We estimated the proportion of women with MBL < 80 mL from mean MBL data (with spread) and from median MBL data (with spread). These estimated proportions, along with estimates of their standard errors, were then used as inputs for the analysis. Data on the proportion of women with a PBAC score < 100 were also used directly as inputs. When studies reported a mean PBAC (with spread) or a median PBAC (with spread), we estimated the proportion of women with PBAC < 100 and used these estimates, along with estimates of their standard errors, as inputs. Additional file 2 discusses these calculations in more detail.

### Statistical model

A Bayesian statistical model related data on % MBL < 80 mL (or % PBAC < 100) to study, treatment class, and follow-up time. The outcomes of main interest were the estimated percentage of women who achieved MBL < 80 mL for various combinations of treatment class and follow-up time. The model included effects for treatment class, the combination of treatment class and time, study, and the combination of treatment class and study. The presence of a study effect preserved the effect of randomization within study.

The efficacy of treatments in achieving MBL < 80 mL may depend on the baseline severity of HMB, measured as baseline MBL. We adjusted for study-level differences in mean baseline MBL by including this as a covariate in the Bayesian model. Thus, the predicted proportion of women with MBL < 80 mL corresponds to a mean baseline MBL equal to the overall average of the mean baseline MBL data reported by all source studies, 170 mL.

In the specification of the model, *y*_*ijt*_ denotes the number of women with MBL < 80 mL (or PBAC < 100) among the *n*_*ijt*_ in study *i* who were assigned to a treatment in treatment class *j* and were present at follow-up time *t*. The probability model for *y*_*ijt*_ is

$$y_{\mathrm{ijt}}∼\mathrm{Binomial}\left(n_{\mathrm{ijt}},p_{\mathrm{ijt}}\right)$$

and the model for *p*_*ijt*_ is logit-linear:

$$\log\mathrm{it}\left(p_{\mathrm{ijt}}\right)=\alpha_{0}+\eta_{i}+\theta_{\mathrm{ij}}+\gamma_{\mathrm{jt}}$$

where logit(*p*) = log_*e*_(*p*/(1–*p*)), α_0_ is the intercept term, η_*i*_ is the random effect for study *i*, θ_*ij*_ is the random effect for the interaction of treatment class *j* and study *i*, and γ_*jt*_ is the incremental effect for follow-up time *t* specific to treatment class *j*. The model treats studies with multiple follow-up times as having the same values of η_*i*_ and θ_*ij*_ for each observation, but potentially different γ_*jt*_ .

The θ_*ij*_ have a random-effects distribution, conditional on treatment-class-specific parameters:

$$\theta_{\mathrm{ij}}∼\mathrm{Normal}\left(\delta_{j}+\alpha_{1}x_{\mathrm{ij}},\tau_{j}^{2}\right)$$

where δ_*j*_ is the treatment-class effect, *x*_*ij*_ is the baseline mean MBL for the arm in treatment class *j* in study *i* (standardized by subtracting the mean over all combinations of *i* and *j* with non-missing values and dividing by the corresponding standard deviation), α_1_ provides the adjustment for *x*_*ij*_, and $\tau_{j}^{2}$ is the conditional variance of the θ_*ij*_ given δ_*j*_ and α_1 ._

Further, the study effects have a random-effects distribution with variance $\sigma_{\eta }^{2}$:

$$\eta_{i}∼\mathrm{Normal}\left(0,\sigma_{\eta }^{2}\right)$$

The model uses a proper, but weakly informative, prior distribution for all parameters. The prior components for the fixed effects (γ_*jt*_ and δ_*j*_) and the other parameters are:

α_0_ ~ Normal (0, 10^4^)

γ_*jt*_ ~ Normal (0, 100)

δ_*j*_ ~ Normal (0, 10^4^), independent

α_1_ ~ Uniform (-5, 5)

τ_*j*_ ~ Uniform (0, 100), independent

$$1/\sigma_{\eta }^{2}$$

~ Gamma (0.1, 0.1)

These prior components are conventional choices. A uniform prior distribution on a standard deviation such as τ_*j*_ is recommended by Gelman and Hill [20].

Missing values of baseline mean MBL were considered to be missing at random [21]. A prior distribution component was used for the missing standardized mean MBL values:

$$x_{\mathrm{ij}}∼\mathrm{Normal}\left(0,1\right)$$

Importantly, although the prior distributions on the missing values are centered at 0, other information in the model moves posterior inferences away from 0.

### Implementation

Estimates of efficacy and all model parameters were obtained through Markov chain Monte Carlo (MCMC) simulation from the posterior distributions. The MCMC simulation for the MTC was implemented using OpenBUGS software (version 3.1.2) [22]. (Three parallel MCMC simulations were run for a burn-in period of 60,000 iterations, after which 60,000 iterations were saved for posterior summaries.) Convergence was assessed through trace plots of several model parameters and a plot of the Gelman-Rubin diagnostic as modified by Brooks and Gelman [23].

## Results

### Literature search

A total of 355 articles met the inclusion and exclusion criteria for efficacy. The review of the full text excluded 310 articles for the reasons listed in Additional file 1 The majority of the excluded articles did not report measures of efficacy suitable for the analysis (i.e., MBL or the PBAC score of Higham et al. [18]).

After all inclusion and exclusion criteria were applied, a total of 45 efficacy studies remained: 32 RCTs and 13 observational studies. The present analysis used only the RCTs. Assessment of the literature published after 2009 found 2 additional RCTs and a later article on one of the initial 32. Thus, efficacy data were available from a total of 34 RCTs; a table summarizing characteristics of the studies is available upon request.

### Evidence network

**Figure 2:**
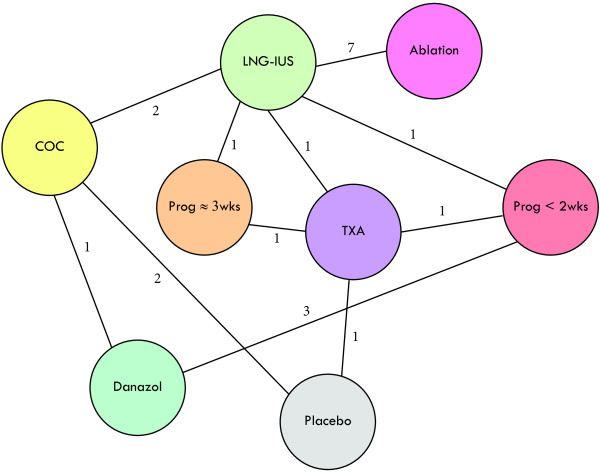
Evidence network of RCTs for MBL in HMB.

Among the 34 RCTs, the most studied treatment classes were ablation (16 RCTs) and LNG-IUS (11 RCTs). Figure 2 shows the treatment classes and direct comparisons that comprised the evidence network. The total number of direct comparisons between treatment classes, 21, differs from the number of RCTs, because 9 studies compared two types of ablation, 1 study had three arms (progestogens administered for less than 2 weeks out of 4 during the menstrual cycle and two regimens of danazol), and 5 studies evaluated treatments not of interest for this study.

The network is thinly connected: only two pairs of treatment classes have more than two direct comparisons, and seven have only one. Only at 3 months are all 8 treatment classes connected (Table 1). At other follow-up times, the number of treatment classes involved in any direct comparison ranges from 7 (at 1 month) to 2 (at 9, 24, and 36 months); at 1 month the network separates into three disjoint components. The trial of LNG-IUS and TXA reported efficacy for the two arms at disjoint times (3, 6, and 12 months for LNG-IUS, and 2 months for TXA) and hence did not provide any direct comparison.

**Table 1:** Comparisons in the evidence network: Number of RCTs that made the comparison, total number of patients in those RCTs, and number of direct comparisons by follow-up time

		**Total N**	**Number of Comparisons**							
			**Follow-up Time (Months)**							
**Comparison**	**RCTs**		**1**	**2**	**3**	**6**	**9**	**12**	**24**	**36**
Ablation & LNG-IUS	7	422			1	2		7	2	1
COC & Danazol	1	24		1						
COC & Placebo	2	355	2	2	2	2				
COC & LNG-IUS	2	91			1	1	1	2		
Danazol & Prog < 2 wks	3	72	1	1	3					
Placebo & TXA	1	166			1					
LNG-IUS & Prog < 2 wks	1	162			1	1				
LNG-IUS & Prog ~ 3 wks	1	38	1		1					
LNG-IUS & TXA	1	31								
Prog < 2 wks & TXA	1	46	1	1						
Prog ~ 3 wks & TXA	1	94			1					

The total number of patients for a given direct comparison (based on the maximum number reported, usually at baseline) was often modest: 4 of the comparisons had fewer than 50 patients, and the largest number was 422.

### Efficacy measures

The data on efficacy had a variety of forms: 11 studies reported the proportion of women achieving MBL < 80 mL; 5 studies reported mean and standard deviation of MBL, and 3 reported median and minimum and maximum of MBL; 11 studies reported the proportion of women with PBAC < 100, 8 studies reported mean and standard deviation of PBAC, 2 reported median and quartiles of PBAC, and 1 reported median and minimum and maximum of PBAC. Several studies reported more than one form, even different forms at different follow-up times for the same arm. The data extraction gave preference to the proportion of women with MBL < 80 mL over summary statistics for MBL when both were available, and similarly for PBAC. For four studies [24-27], we obtained data on the proportion of women with MBL < 80 mL or PBAC < 100 from a clinical study report or a subsequent analysis.

Only 15 studies reported a mean of MBL at baseline, ranging from 90.3 mL to 300 mL. The average (over study arms with non-missing data) was 169.64 mL.

### Posterior summaries

In line with convention we report posterior medians with 95% credible intervals (CrI) (whose endpoints are the 2.5 and 97.5 percentage points of the posterior distribution). Table 2 presents these estimates of efficacy at 3 months (the interval at which patients in the economic model were evaluated). Because the statistical model adjusts for the baseline mean of MBL, those estimates are stated at a baseline MBL of 169.64 mL.

**Table 2:** Efficacy estimates for the 8 treatment classes at 3 months

**Treatment**	**% MBL < 80 mL (95% CrI)**
LNG-IUS	87.5 (77.6-93.9)
Ablation	81.6 (5.6-99.7)
Danazol	65.8 (33.6-93.0)
Prog ≈ 3wks	63.6 (20.2-95.0)
COC	63.4 (44.3-78.7)
TXA	48.2 (28.8-65.4)
Placebo	17.7 (7.9-36.1)
Prog < 2wks	14.2 (3.7-41.7)

Posterior median with 95% CrI.

Based on available data, estimates after 3 months of treatment indicate the following descending order of efficacy (posterior median): LNG-IUS and endometrial ablation with comparably high response rates (87.5% and 81.6% of women achieving MBL < 80 mL, respectively), followed by danazol (65.8%), progestogens given for close to 3 weeks out of 4 during the menstrual cycle (63.6%), COCs (63.4%), and TXA (48.2%). Progestogens administered for less than 2 weeks out of 4 (14.2%) were not better than placebo (17.7%).

The widths of the 95% credible intervals range from 16 percentage points for LNG-IUS to 94 percentage points for ablation. Among the other six treatment classes, four widths range from 28 to 38 percentage points, one is 59, and the other is 75. Thus, most estimates had substantial uncertainty. Only LNG-IUS and COCs had credible intervals that did not overlap the interval for placebo.

## Discussion

The MTC framework was convenient for synthesizing available evidence and estimating % MBL < 80 mL at various follow-up times, but our focus was not on comparing treatment classes, nor on comparing treatments within classes (the data generally were not sufficient). The validity and reliability of the evidence for compounds within the same class (e.g., COCs) varies among studies, and pooled estimates for treatment classes may not account for some variation in efficacy within the class. The main aim was to estimate efficacy at a follow-up time of 3 months, with corresponding credible intervals, as inputs in a microsimulation economic model evaluating the relative cost and health impact of the eight treatment classes. The analysis produced posterior median estimates of % MBL < 80 mL that plausibly reflect the current evidence: a high level of efficacy for LNG-IUS and endometrial ablation [28] and somewhat lower efficacy for oral treatments. LNG-IUS and ablation, however, are designed for long-term (1 year or longer) reduction of menstrual bleeding. For women who prefer oral treatments and reversible contraception, COCs are an appropriate option.

Our evidence synthesis used a Bayesian framework, rather than a frequentist analysis, because Bayesian methods for indirect comparisons and MTCs are much more fully developed, offer greater flexibility in handling the special features of our data (e.g., availability of both direct and indirect evidence for some comparisons, uncertainty of % MBL < 80 mL and % PBAC < 100 estimated from summary statistics, accounting for missing data), and avoid problems associated with inverse-variance weighting based on estimated variances, such as bias and confidence-interval coverage that departs substantially from the nominal value [29]. However, the use of a treatment-class effect, rather than treatment-specific effects within a class, may not fully account for some variation in efficacy between interventions within the same class.

A number of systematic reviews have synthesized the evidence for subsets of the treatment classes, and a network meta-analysis compared six second-generation endometrial ablation techniques (primarily with the class of first-generation hysteroscopic devices as the reference treatment) [30]. However, this is the first study that has combined data on eight treatment options for heavy menstrual bleeding.

The systematic reviews comparing LNG-IUS and endometrial ablation have produced inconsistent conclusions. Marjoribanks et al. [31] concluded that resection or ablation was more effective than LNG-IUS at controlling bleeding at 1 year, but the evidence for longer-term effects was inconclusive. Lethaby et al. [12], addressing the same comparison from the opposite perspective, also found that LNG-IUS produced a smaller mean reduction in MBL (the primary endpoint) than ablation and progestogen side effects, but with no evidence of a difference in satisfaction or perceived quality of life between LNG-IUS and ablation. In contrast, Kaunitz et al. [32] subsequently used trial-level means and standard deviations of PBAC scores (their primary endpoint) from six RCTs to compare LNG-IUS and ablation. They concluded that at 6, 12, and 24 months, LNG-IUS was at least as effective as ablation in reducing MBL.

Middleton et al. [33] identified 30 RCTs that compared pairs of treatments from the classes first-generation ablation, second-generation ablation, LNG-IUS, and hysterectomy, and assembled individual patient data (IPD) from 17 of them. The primary outcome measure was satisfaction, but they also analyzed available data on MBL. Having IPD allowed them “to use previously unreported data, improve the assessment of study quality, standardize outcome measures, undertake intention-to-treat analysis, and use optimal analytical methods.” In their analysis LNG-IUS and endometrial ablation were comparable, but the authors remarked on uncertainty from small sample sizes in studies of LNG-IUS.

The findings of these systematic reviews of direct comparisons add support to the indication from our analysis that ablation is an effective treatment for HMB.

Lethaby et al. also evaluated a number of pharmacological therapies for HMB in a series of systematic reviews. They concluded that oral progestogens administered only during the luteal phase were less effective at reducing MBL than tranexamic acid, danazol, and LNG-IUS. Progestogens taken between day 5 and day 26 of the cycle, however, significantly reduced MBL from baseline, but were less effective than LNG-IUS [10,12]. Danazol seemed to be more effective than placebo, progestogens, or COC, but confidence intervals were wide (based on pooled data from nine RCTs) [34]. Tranexamic acid was more effective than placebo and luteal-phase progestogens at reducing MBL [11]. An additional review of the effect of COCs on MBL by Farquhar and Brown [9] located only one cross-over study of 45 women, which found no significant difference in MBL between COC and danazol or non-steroidal anti-inflammatory drugs. Marjoribanks et al. [31] concluded that the results of these reviews “suggest that the LNG-IUS system provides a better alternative to surgery than oral medication. Levels of satisfaction and quality of life reported by women with an LNG-IUS system are similar to those in women who have undergone transcervical endometrial ablation or balloon ablation. Surgical methods are significantly more effective in reducing bleeding at one year, but studies with longer follow up did not show an ongoing advantage for surgery.”

These systematic reviews therefore agree with the estimates from our MTC that LNG-IUS and ablation are the most effective of the treatments studied at reducing MBL, that progestogens given for less than 2 weeks out of 4 during the menstrual cycle are least effective, and that danazol, progestogens given for close to 3 weeks out of 4, and tranexamic acid also showed efficacy. Our MTC was able to produce stronger evidence to support the use of COCs in HMB, largely by identifying studies that were not published at the time of the review by Farquhar and Brown [9]. The previous systematic reviews found no direct-comparison studies for oral progestogens versus placebo, danazol versus TXA, danazol versus LNG-IUS, or LNG-IUS versus placebo, but our MTC suggested comparative efficacy for these treatments.

As mentioned in the introduction, we encountered several methodological challenges. First, the limited amount of data available contributed to the substantial uncertainty in most of our estimates of efficacy. Most of the studies had fairly modest sample sizes (median number of patients per arm 33, range 9 to 164). Also, as indicated earlier, studies varied greatly in the measures used and in study designs; 21 direct comparisons were spread among 8 treatment classes, and only one pair of treatment classes had more than 3 direct comparisons (Figure 1).

Further, the small number of follow-up times that were common across treatment classes (Table 1) increased uncertainty. Some variation in follow-up time is a consequence of the nature of the treatment classes. For example, for endometrial ablation, follow-up times of 6, 12, and 24 months are common. Among the seven studies that compared ablation and LNG-IUS, only one reported efficacy at 3 months. Thus, the wide credible interval at 3 months (94 percentage points) is not surprising. For other treatment classes (e.g., danazol and TXA), follow-up times of 1, 2, 3, and 6 months are more appropriate. The apparent lack of consensus on follow-up times among researchers studying a particular treatment class presents a challenge for evidence synthesis.

Estimation of % MBL < 80 mL (or % PBAC < 100) from summary statistics for MBL (or PBAC) introduced additional uncertainty, and each of the three distinct sets of summary statistics (mean and standard deviation, median and minimum and maximum, and median and quartiles) required a separate procedure for estimating % MBL < 80 mL or % PBAC < 100 (and a further, more-complicated procedure for estimating the standard error of the estimate). We wanted, however, to use as much of the available evidence as possible. In some articles we were unable to extract the same measure of efficacy at all follow-up times, or even for both treatments. Investigators showed little consensus on the measures of efficacy to report, with no convergence of approach over time. Future researchers can facilitate meta-analyses and MTCs by reporting outcomes in a more consistent way and in sufficient detail (e.g., in a supplemental file, available online) for secondary analysis.

The greater use of measures based on PBAC scores may reflect a shift away from the burden that use of MBL places on trial participants (who must collect their sanitary material for laboratory analysis). We used only data based on the PBAC score developed by Higham et al. [18] because it was much more common in the articles that we encountered than scores based on other pictorial charts. The validity of both PBAC and the alkaline hematin method requires consistent use of the specific validated sanitary materials. Deviations from this requirement may affect estimates of efficacy; but they are difficult to measure and are not reported in the studies’ results, adding to unexplained variation and uncertainty of the estimates.

In some MTC meta-analyses it may be advantageous to include RCTs that evaluated only one, or even none, of the treatments of interest [35]. When comparisons of efficacy are the focus, the network of evidence would then ordinarily include all the treatments evaluated in those RCTs. Five of the RCTs in our data evaluated treatments that were not considered in the microsimulation model, and we did not include data from the other arms of those RCTs. In four of the five, the other treatment was mefenamic acid (which is no longer considered a strong treatment option), and in the fifth it was hysterectomy.

Several areas would benefit from attention in future work: the effect of including additional treatment classes in the evidence network, including RCTs that reported outcomes based on other pictorial charts, incorporating results from observational studies, and synthesizing evidence on patient-focused outcomes such as satisfaction and health-related quality of life.

## Conclusions

Synthesis of the evidence in an MTC framework yielded plausible estimates of % MBL < 80 mL at 3 months for the eight treatment classes. LNG-IUS and endometrial ablation had the highest efficacy, but the 95% credible interval for ablation was very wide. The widths of the credible intervals reflect the various sources of uncertainty taken into account in the Bayesian model. Thus, more evidence is needed, particularly for the classes of oral treatments.

Besides the sparse and fragmented nature of the evidence network, an important source of uncertainty arose from having to estimate % MBL < 80 mL or % PBAC < 100 from summary statistics. Consistent reporting of an outcome measure, reflecting a consensus of investigators studying HMB, could do much to reduce this uncertainty.

## Abbreviations

CEBM: Centre for Evidence Based Medicine; COC: Combined oral contraceptive; CrI: Credible interval; GRADE: Grading of Recommendations Assessment, Development and Evaluation; HMB: Heavy menstrual bleeding; IPD: Individual patient data; LNG-IUS: Levonorgestrel-releasing intrauterine system; MBL: Menstrual blood loss; MCMC: Markov chain Monte Carlo; MTC: Mixed treatment comparison; NHS: National Health Service; NICE: National Institute for Health and Clinical Excellence; PBAC: Pictorial blood-loss assessment chart; RCT: Randomized controlled trial; TXA: Tranexamic acid.

## Competing interests

This study was supported by Bayer Pharma AG, Berlin, Germany. DCH, MEG, RW, and RG served as contractors for Bayer; RW, DCH, and RG were employees of United BioSource Corporation when the research was executed, and MEG is an employee of Boston University. AF is an employee of Bayer Pharma AG.

## Authors’ contributions

DCH, AF, MEG, and RG contributed to study design and execution. DCH drafted the manuscript. RG coordinated the study and reviewed the manuscript. AF and MEG reviewed and commented on manuscript drafts. RW participated in study coordination and helped to draft the manuscript. All authors read and approved the final manuscript.

## Supplementary Material

Additional file 1 Inclusion and Exclusion Criteria for Full Literature Search and Inclusion and Exclusion Criteria for Efficacy. Click here for file

Additional file 2 Estimates of % MBL <80 mL and % PBAC <100 and Their Uncertainty. Click here for file
