# Supplementary material for: Use of mixed-treatment-comparison methods in estimating efficacy of treatments for heavy menstrual bleeding
Source: Eur J Med Res. 2013 Jun 21;18(1):17. doi: 10.1186/2047-783X-18-17 (PMC3698104; doi:10.1186/2047-783X-18-17)
Supplement: Additional file 1 — Inclusion and Exclusion Criteria for Full Literature Search and Inclusion and Exclusion Criteria for Efficacy. [file 2047-783X-18-17-S1.docx]

# Additional File 1

# Inclusion and Exclusion Criteria (Full Literature Search)

| **Inclusion Criteria** |
| --- |
| **Population/Indication/Study Design (must meet all):** |
| Women, age ≥ 18 years |
| HMB, DUB, AUB, menorrhagia, heavy, excessive, or prolonged bleeding |
| Menorrhagia, metrorrhagia, menometrorrhagia, intermenstrual bleeding, polymenorrhea,  oligomenorrhea or amenorrhea or |
| Interventional or observational study |
| Reviews and guidelines |
| **Content (must meet at least one):** |
| **Interventions (must meet at least one)** |
| Hormonal contraceptives (oral and long acting reversible) |
| Other pharmaceutical treatments, such as danazol, tranexamic acid, Gonadotropin-releasing hormone, NSAIDs |
| LNG-IUS/Mirena |
| Ablation |
| Hysterectomy (vaginal, abdominal, laparascopically assisted) |
| Other (Office biopsy, D&C) |
| **Outcomes (must meet at least one):** |
| Epidemiology (prevalence, incidence, other data stratified by demographic characteristics) |
| Measurement of MBL |
| Clinical efficacy/effectiveness (objective or patient-reported) |
| Treatment failure |
| Patients' preferences for/satisfaction with interventions for the management of HMB |
| Patient-reported heath-related quality of life including utility values |
| Burden associated with the disease |
| Change in outcomes following a therapeutic intervention |
| Economic burden of the disease (e.g., cost of illness) |
| Utilization of any treatment-related resources |
| Cost-effectiveness of HMB interventions |
| Economic outcomes of treatments/interventions for HMB including evaluations of different care delivery methods/settings of care |
| **Exclusion Criteria** |
| Studies that evaluated any aspects of: Vaginal bleeding related to pregnancy/post-partum Malignancies or organic structural uterine/endometrial changes Inherited bleeding disorders or other systemic diseases causing menstrual bleeding disturbances |
| Studies that *only* assessed contraceptive effects of hormonal pharmaceutical therapies |
| Articles not written in English |

# Inclusion and Exclusion Criteria—Efficacy

## *Inclusion Criteria*

#### Participants

- Non-menopausal human females aged 19+ diagnosed with any of the following and/or articles including the following key words or topics:
- Dysfunctional uterine bleeding
- Abnormal uterine bleeding
- Excessive uterine bleeding
- Heavy uterine bleeding
- Heavy and/or prolonged menstrual bleeding (hpmb)
- Prolonged heavy period
- Menorrhagiatechnii
- Metrorrhagia
- Menometrorrhagia
- Intermenstrual bleeding
- Polymenorrhea
- Oligomenorrhea
- Hypermenorrhea

#### Interventions

- Inactive treatment (placebo)
- Active treatment

#### Comparisons

- Drug treatments (each considered as a separate class):
- Combined oral contraceptives (COC)
- Tranexamic acid (TXA)
- Oral progesterone/progestogens
- Injectable progesterones/progestogens
- Progesterones/progestogens administered during luteal phase of menstrual cycle
- Progesterones/progestogens administered during non-luteal phase of menstrual cycle
- Danazol
- Progesterone-Intrauterine systems (Prog-IUS), including the levonorgestrel-releasing intrauterine system and Progestasert®
- Placebo
- Surgical treatments
- Endometrial ablation/resection (any type)

#### Outcomes

- Mean MBL
- Median MBL
- % MBL < 80 mL
- Mean PBAC
- Median PBAC
- % PBAC < 100

#### Study Design

- Randomized controlled trial
- Prospective observational study
- Retrospective observational study

#### Date of Publication

- 01/01/1966 to 2009

#### Language

- English

## *Exclusion Criteria*

- Studies that evaluated any aspect of:
- Endometriosis
- Adenomyosis
- Polycystic ovary syndrome
- Vaginal bleeding related to pregnancy/post-partum
- Malignancies or organic structural uteral/endometrial changes
- Inherited bleeding disorders or other systemic disease causing menstrual bleeding disturbances
- Oligomenorrhea (except as an outcome of treatment for HMB)
- Metrorrhagia (except as an outcome of treatment for HMB)
- Amenorrhea (except as an outcome of treatment for HMB)
- Any other uterine bleeding disorder of an organic nature, injury, structural defect, or identified hormonal or genetic disorder
- Studies that did not report a measure of spread around mean or median
- Cross-over studies that do not have a “wash-out” period of at least 2 months or that do not report outcome data separately for each treatment period
- Studies that used a non-standard PBAC (i.e., studies that did not use the PBAC developed by Higham et al., 1990)
- Studies that report efficacy outcomes as categories such as “menorrhagia” and “amenorrhagia” and do not define these categories in terms of mL of menstrual fluid lost
- Studies in which patients were concomitantly using an anticoagulant (e.g., warfarin)
- Studies in which patients were concomitantly using an anti-diuretic
- Studies whose sample had a mean MBL < 80 mL at baseline (indicating that the average patient was a “responder” before receiving the treatment)
- Studies in which ≥ 50% of women did not have menorrhagia (defined as MBL > 80 mL) at baseline

# Reasons for Exclusion in Review of Full Text

| **Reason for Exclusion** | **Total** |
| --- | --- |
| Did not assess MBL (mL) or PBAC | 191 |
| Did not assess efficacy | 42 |
| Overview or review article | 28 |
| Definition of population not compatible | 16 |
| MBL or PBAC data not usable | 14 |
| Used PBAC score other than Higham | 10 |
| Not RCT or observational study | 4 |
| Article not available for purchase | 3 |
| Ablation procedure was performed with a non-working device in 21 patients | 1 |
| Meta-analysis | 1 |
| **Total** | **310** |

## Articles from Which Data Were Extracted

1. Bonnar J, Sheppard BL. **Treatment of menorrhagia during menstruation: randomised controlled trial of ethamsylate, mefenamic acid, and tranexamic acid**. *British Medical Journal* 1996, **313**:579-82.
2. Brun JL, Raynal J, et al. **Cavaterm thermal balloon endometrial ablation versus hysteroscopic endometrial resection to treat menorrhagia: the French, multicenter, randomized study**. *Journal of Minimal Invasive Gynecology* 2006, **13**:424-30.
3. Busfield RA, Farquhar CM, et al. **A randomised trial comparing the levonorgestrel intrauterine system and thermal balloon ablation for heavy menstrual bleeding**. *BJOG: an International Journal of Obstetrics and Gynaecology* 2006, **113**:257-63.
4. Cameron IT, Haining R, et al. **The effects of mefenamic acid and norethisterone on measured menstrual blood loss**. *Obstetrics and Gynecology* 1990, **76**:85-88.
5. Cooper J, Gimpelson R, et al. **A randomized, multicenter trial of safety and efficacy of the NovaSure system in the treatment of menorrhagia**. *The Journal of the American Association of Gynecologic Laparoscopists* 2002, **9**:418-428.
6. Corson SL, Brill AI, et al. **One-year results of the vesta system for endometrial ablation**. *The Journal of the American Association of Gynecologic Laparoscopists* 2000, **7**:489-497.
7. Crosignani PG, Vercellini P, et al. **Levonorgestrel-releasing intrauterine device versus hysteroscopic endometrial resection in the treatment of dysfunctional uterine bleeding**. *Obstetrics and Gynecology* 1997, 90:257-263.
8. Dockeray CJ, Sheppard BL, et al. **Comparison between mefenamic acid and danazol in the treatment of established menorrhagia**. *British Journal of Obstetrics and Gynaecology* 1989, **96**:840-844.
9. Duleba AJ, Heppard MC, et al. **A randomized study comparing endometrial cryoablation and rollerball electroablation for treatment of dysfunctional uterine bleeding**. *The Journal of the American Association of Gynecologic Laparoscopists* 2003, **10**:17-26.
10. Dunphy BC, Goerzen J, et al. **A double-blind randomised study comparing danazol and medroxyprogesterone acetate in the management of menorrhagia**. *Journal of Obstetrics and Gynaecology* 1998, **18**:553-555.
11. Endrikat J, Shapiro H, et al. **A Canadian, multicentre study comparing the efficacy of a levonorgestrel-releasing intrauterine system to an oral contraceptive in women with idiopathic menorrhagia**. *Journal of Obstetrics and Gynaecology Canada* 2009, **31**:340-347.
12. Fraser IS, McCarron G. **Randomized trial of 2 hormonal and 2 prostaglandin-inhibiting agents in women with a complaint of menorrhagia**. *The Australian & New Zealand Journal of Obstetrics & Gynaecology* 1991, **31**:66-70.
13. Fraser IS, Römer T, Parke S, Zeun S, Mellinger U, Machlitt A, Jensen JT. **Effective treatment of heavy and/or prolonged menstrual bleeding with an oral contraceptive containing estradiol valerate and dienogest: a randomized double-blind Phase III trial.** *Human Reproduction.* 2011, **26**(10):2698-2708.
14. Higham JM, Shaw RW. **A comparative study of danazol, a regimen of decreasing doses of danazol, and norethindrone in the treatment of objectively proven unexplained menorrhagia**. *American Journal of Obstetrics and Gynecology* 1993, **169**:1134-1139.
15. Hurskainen R, Teperi J, et al. **Quality of life and cost-effectiveness of levonorgestrel-releasing intrauterine system versus hysterectomy for treatment of menorrhagia: a randomised trial**. *Lancet* 2001, **357**:273-277.
16. Hurskainen R, Teperi J, et al. **Clinical outcomes and costs with the levonorgestrel-releasing intrauterine system or hysterectomy for treatment of menorrhagia: randomized trial 5-year follow-up**. *Journal of the American Medical Association* 2004, **291**:1456-1463.
17. Irvine GA, Campbell-Brown MB, et al. **Randomised comparative trial of the levonorgestrel intrauterine system and norethisterone for treatment of idiopathic menorrhagia**. *British Journal of Obstetrics and Gynaecology* 1998, **105**:592-598.
18. Istre O, Trolle B. **Treatment of menorrhagia with the levonorgestrel intrauterine system versus endometrial resection**. *Fertility and Sterility* 2001, **76**:304-309.
19. Jensen JT, Parke S, Mellinger U, Machlitt A, Fraser IS. **Effective treatment of heavy menstrual bleeding with estradiol valerate and dienogest: A randomized controlled trial.** *Obstet Gynecol*. 2011;**117**:777-787.
20. Kaunitz AM, Bissonnette F, et al. **Levonorgestrel-releasing intrauterine system or medroxyprogesterone for heavy menstrual bleeding: a randomized controlled trial**. *Obstet Gynecol* 2010, **116**(3):625-632.
21. Kittelsen N, Istre O. **A randomized study comparing levonorgestrel intrauterine system (LNG IUS) and transcervical resection of the endometrium (TCRE) in the treatment of menorrhagia: preliminary results**. *Gynaecological Endoscopy* 1998, **7**:61-65.
22. Kriplani A, Kulshrestha V, et al. **Role of tranexamic acid in management of dysfunctional uterine bleeding in comparison with medroxyprogesterone acetate*.*** *Journal of Obstetrics and Gynaecology* 2006, **26**:673-678.
23. Lukes AS, Moore KA, et al. **Tranexamic acid treatment for heavy menstrual bleeding: a randomized controlled trial.** *Obstet Gynecol* 2010, **16**(4):865-875.
24. Malak K, Shawki O. **Management of menorrhagia with levonorgestrel intrauterine system versus endometrial resection**. *Gynecological Surgery* 2006, **3**:275-280.
25. Meyer WR, Walsh BW, et al. **Thermal balloon and rollerball ablation to treat menorrhagia: a multicenter comparison**. *Obstetrics & Gynecology* 1998, **92**:98-103.
26. Milsom I, Andersson K, et al. **A comparison of flurbiprofen, tranexamic acid, and a levonorgestrel-releasing intrauterine contraceptive device in the treatment of idiopathic menorrhagia**. *American Journal of Obstetrics and Gynecology* 1991, **164**:879-883.
27. Perino A, Castelli A, et al. **A randomized comparison of endometrial laser intrauterine thermotherapy and hysteroscopic endometrial resection**. *Fertility and Sterility* 2004, **82**:731-734.
28. Preston JT, Cameron IT, et al. **Comparative study of tranexamic acid and norethisterone in the treatment of ovulatory menorrhagia**. *British Journal of Obstetrics and Gynaecology* 1995, **102**:401-406.
29. Rauramo I, Elo I, et al. **Long-term treatment of menorrhagia with levonorgestrel intrauterine system versus endometrial resection**. *Obstetrics and Gynecology* 2004, **104**:1314-1321.
30. Reid PC, Virtanen-Kari S. **Randomised comparative trial of the levonorgestrel intrauterine system and mefenamic acid for the treatment of idiopathic menorrhagia: a multiple analysis using total menstrual fluid loss, menstrual blood loss and pictorial blood loss assessment charts**. *BJOG: an International Journal of Obstetrics and Gynaecology* 2005, **112**:1121-5.
31. Sambrook AM, Cooper KG, et al. **Clinical outcomes from a randomised comparison of Microwave Endometrial Ablation with Thermal Balloon endometrial ablation for the treatment of heavy menstrual bleeding**. *BJOG: an International Journal of Obstetrics and Gynaecology* 2009, **116**:1038-45.
32. Shabaan MM, Zakherah MS, et al. **Levonorgestrel-releasing intrauterine system compared to low dose combined oral contraceptive pills for idiopathic menorrhagia: a randomized clinical trial**. *Contraception* 2011, **83**(1):48-54.
33. Soysal ME, Soysal SK, et al. **Thermal balloon ablation in myoma-induced menorrhagia under local anesthesia**. *Gynecologic and Obstetric Investigation* 2001, **51**:128-133.
34. Soysal M, Soysal S, et al. **A randomized controlled trial of levonorgestrel releasing IUD and thermal balloon ablation in the treatment of menorrhagia**. *Zentralblatt für Gynäkologie* 2002, 124:213-219.
35. Vercellini P, Oldani S, et al. **Randomized comparison of vaporizing electrode and cutting loop for endometrial ablation.** *Obstetrics and Gynecology* 1999, **94**:521-527.
