# Supplementary material for: Use of mixed-treatment-comparison methods in estimating efficacy of treatments for heavy menstrual bleeding
Source: Eur J Med Res. 2013 Jun 21;18(1):17. doi: 10.1186/2047-783X-18-17 (PMC3698104; doi:10.1186/2047-783X-18-17)
Supplement: Additional file 2 — Estimates of % MBL <80 mL and % PBAC <100 and Their Uncertainty. [file 2047-783X-18-17-S2.docx]

## Additional File 2

## *Estimates of % MBL < 80mL and % PBAC < 100 and Their Uncertainty*

When a trial reported summary statistics for MBL or PBAC, it was possible in most instances to estimate % MBL < 80 mL or % PBAC < 100 from those statistics, but with less precision than if those percentages had been reported. By analyzing patient-level data from two clinical trials (Fraser et al. 2011, Jensen et al. 2011), we determined that the distribution of MBL was well approximated by a lognormal distribution. Also, data digitized from a figure in Higham et al. (1990) showed that the distribution of PBAC scores was approximately lognormal. By estimating the parameters of the lognormal distribution from available summary statistics, we obtained an estimate of % MBL < 80 mL (or % PBAC < 100). We also estimated the standard error of that estimate.

By definition, if *Y* has a 2-parameter lognormal distribution, the logarithm of *Y* follows a normal distribution. That is,

$\log_{e}(Y)$ ~ $N(\mu,\sigma^{2})$

for some constant $\mu$ and some positive constant $\sigma$. When an article reported the mean, , and standard deviation, s, we based our estimates of and on those (without being able to take into account 0s or possible outliers). The formulas for the estimates are

and

.

When an article reported the median and the quartiles of MBL, we preferred those to the mean and the standard deviation, because the median and quartiles are generally less affected by outliers and 0s. Also, estimation of , the mean of the assumed normal distribution of log*_e_*(MBL), from the median of MBL is easier than from the mean, because the mean and median of a normal distribution are equal. If we denote the median of MBL by *M*, then

.

Estimation of from the quartiles is straightforward. If we denote the quartiles of MBL by and , we transform them to the quartiles of log*_e_*(MBL) by taking logs: and . Then, since and are generally the result of interpolation, it is satisfactory to estimate by dividing the interquartile range in the log scale by the corresponding value from the standard normal distribution, regardless of the sample size:

.

Then the estimated probability of MBL < 80 mL is

,

where denotes the cumulative distribution function of the standard normal distribution and 4.382 is the natural logarithm of 80. We interpret this estimated probability as the proportion of patients with MBL < 80 mL in a population of patients whose mean and standard deviation equal those observed in the sample.

Other combinations of summary statistics, each with corresponding formulas for and , included the median and minimum and maximum.

We used a similar approach to estimate % PBAC < 100 when an article instead reported summary statistics for PBAC scores.

The uncertainty in the underlying estimates and the transformations involved both contribute to the uncertainty of the estimates of % MBL < 80 mL and % PBAC < 100. Thus, we derived an estimate of the variance of the estimate of % MBL < 80 mL or % PBAC < 100 for each combination of summary statistics. For example, in the estimate $\hat{P}\{\text{MBL}<\text{80 mL}\}=\Phi((\log_{e}80-\hat{\mu})/\hat{\sigma})$ the uncertainty in $\hat{P}$ comes from the sampling variability in $\hat{\mu}$ and$\hat{\sigma}$.

Because , the cumulative distribution function of the standard normal distribution, is a non-linear function, we applied a standard technique known as the delta method to obtain an approximate estimate of the variance of. Our application of the delta method took into account the joint sampling variability of $\hat{\mu}$ and $\hat{\sigma}$, but a simpler version illustrates the basic idea. Suppose that a statistic *T* has expected value $\theta$ and that we estimate $g(T)$, where *g* is a function that satisfies certain (relatively mild) conditions. The first-order Taylor-series expansion of *g* about $\theta$ (omitting terms of second and higher order) yields the approximation

$g(t)\approx g(\theta)+g'(\theta)(t-\theta)$,

where $g'(\theta)$ denotes $dg/dt$ evaluated at $\theta$. Then the expected value of $g(T)$ is (approximately)$g(\theta)$ , and the variance of $g(T)$ is (approximately)

$\mathrm{var}[g(T)]\approx[g'(\theta)]^{2}\mathrm{var}(T)$.

In general, the value of $\theta$ is unknown, so one customarily substitutes the estimate, *T*, and calculates

$\mathrm{var}[g(T)]\approx[g'(T)]^{2}\mathrm{var}(T)$.

The Bayesian model uses $\hat{P}$ in the logit scale.

To account for the uncertainty in the estimates of % MBL < 80 mL or % PBAC < 100 obtained from summary statistics, the Bayesian model incorporated the following components. For MBL, let *p* be the probability that MBL < 80 mL and $\hat{p}$ be the corresponding estimate. The model used

logit($\hat{p}$) ~ Normal(logit(p), *s*^2^),

where *s*^2^ is the estimated variance of logit($\hat{p}$). We made a similar calculation for estimates of the probability that PBAC < 100.
